# A Remarkable Fall

**Published:** 1870-05

**Authors:** Henry Shimer

**Affiliations:** Mount Carroll, Ill.


### A REMARKABLE FALL.

MOUNT CARROLL, ILL., *March 27, 1870.*

A Miss A. B., age 13, while sliding on the snow, February 5, accidentally ran over a perpendicular precipice of 54 feet (measured), alighting on the ice of the mill-dam below. The sled was broken. She was soon found and taken home, soon recovering sensibility; no bones were broken.

I was called in consultation with Dr. Etter and Dr. Miller, on the following day. There was some soreness in the gluteal regions and about the pelvis; the bladder was paralized, so as to require the use of the catheter three times. Temperature 101°. She made a complete recovery in two weeks. Had she been in the same condition from a fall of six feet, the friends would have seen no cause for alarm.

HENRY SHIMER,

RELAPSING FEVER IN EDINBURGH.—This fever has at last broken out in Edinburgh, in two different localities, without, we believe, any contagious influence being traceable in either case. The history of this outbreak, carefully recorded, as we hope it shall be, must form one of the most interesting episodes in the modern history of fever, as well as a not unimportant contribution to the vexed question of change of type.—*Edinburgh Med. Journal, March, 1870.*

